# Editorial: MicroRNA-related polymorphisms in infectious and inherited diseases

**DOI:** 10.3389/fgene.2023.1192457

**Published:** 2023-04-11

**Authors:** Yusuf Tutar, Dilek Pirim, Aftab Ali Shah, Antonio C. R. Vallinoto

**Affiliations:** ^1^ Division of Biochemistry, Department of Basic Pharmaceutical Sciences, Hamidiye Faculty of Pharmacy, Istanbul, Turkey; ^2^ Division of Molecular Oncology, Health Sciences Institute, Istanbul, Turkey; ^3^ Personalized Medicine and Immunotherapy Research Center, Istanbul, Turkey; ^4^ Experimental Medicine Application and Research Center, Validebag Research Park, University of Health Sciences, Istanbul, Turkey; ^5^ Institute of Health Science, Department of Translational Medicine, Bursa, Turkey; ^6^ Department of Molecular Biology and Genetics, Bursa Uludag University, Bursa, Turkey; ^7^ Department of Biotechnology, University of Malakand, Chakdara, Pakistan; ^8^ Laboratory of Virology, Institute of Biological Sciences, Federal University of Pará, Belém, Brazil

**Keywords:** miRNA, polymorphism, disease, SNP, GWAS

Recent advances in high-throughput sequencing approaches have revealed a large portion of the human genome encodes non-coding RNA (ncRNA) molecules that may have a key role in gene regulation, epigenetic regulation, and post-transcriptional regulation. Thus, deciphering the function of ncRNAs and the molecular mechanisms underlying their functionality are essential research areas in human genetics. MicroRNAs (miRNAs) are one major group of regulatory ncRNAs of ∼22–24 nucleotides that fine-tune gene regulation at the post-transcriptional level. MiRNA-mediated gene regulation occurs when miRNA interacts with specific complementary sequences of the target RNA and disrupts the translation initiation or elongation process (Tutar et al., 2018; Ghanbarian et al., 2022).

Mature miRNAs are generated from DNA sequences by multiple steps including primary miRNAs (pri-miRNA) and precursor miRNA (pre-miRNA) processing events. MicroRNA-related polymorphisms lying in the miRNA coding DNA sequences or 3’ UTRs of the target miRNA binding site have the potential to affect miRNA expression and mRNA-miRNA interaction by altering miRNA biogenesis and function (Ryan, 2017). Hence, identifying the single nucleotide polymorphisms (SNPs) contributing to disease risk by changing the function of ncRNA and gene expression may provide an innovative approach to translational applications and offer novel treatment approaches in clinical settings. MiRNA-related polymorphisms associated with human diseases form the main basis of this theme issue, yet SNPs affecting RNA modifications were also emphasized in the published articles.

The first topic “*Association of Genetic Variants Affecting microRNAs and Pancreatic Cancer Risk*” overviews miRNA-related polymorphisms associated with the risk of pancreatic cancer. Lu et al. retrieved and re-analyzed GWAS data from the Pancreatic Cancer Cohort Consortium (PanScan I-III) and Pancreatic Cancer Case Control Consortium (PanC4) to select SNPs for testing their associations with pancreatic cancer (PC) in a separate cohort. SNPs located in the miRNA seed regions and 3′UTRs of the target genes were selected for genotyping purposes. Their meta-analysis prioritized four miRNA-related SNPs (rs13246412, rs4977756, rs7985480, and rs2975216) showing convincing associations with the risk of developing PC, and *in silico* analysis revealed their putative effects on the binding site of several miRNAs (Lu et al.).

Another GWAS study pinpoints SNPs related to RNA modifications which yield associations with the risk for spontaneous coronary aortic dissection (SCAD) (Chai et al.). Aortic dissections (AD) are intricate conditions that damage the aorta and a common cause of global morbidity and death. The typical course of aortic dissections starts with a rip in the aortic intima, followed by blood infiltration into the media and separating the mid-membrane that differentiates a true lumen from a false lumen. RNA modification is crucial for numerous biological processes, including regulation of gene expression, mRNA stability, and homeostasis. Aortic dissection may be caused by genetic variations that alter RNA modification. Chai et al. investigated RNA modification-associated SNPs associated with SCAD and identified multiple functionally relevant SNPs that are significantly associated with SCAD. Their results highlight the pivotal role of RNA modifications in the molecular pathogenesis of SCAD.

A unique study on miRNA-related polymorphism is “*Genetic Variants of MicroRNA and DROSHA Genes in Association With the Risk of Tuberculosis in the Amazon Population.*” This study scrutinized the associations between 26 SNPs and tuberculosis to determine whether these SNPs could be risk factors for the disease in the Amazon population (Leal et al.). Their results indicated that the rs10035440 (DROSHA), rs7372209 (miR26-a1), rs1834306 (miR100), rs4919510 (miR608), and rs10739971 (pri-let-7a-1) were significantly associated with high risk, and rs3746444 (miR499) and rs6505162 (miR423) were found to be significantly associated with low risk of developing tuberculosis in the Amazon population. The research provides new insight into the molecular pathogenesis of tuberculosis and may lead to the development of innovative diagnostic techniques for *M. tuberculosis* infection.

The final work by Flowers et al. “*The Role of Racial and Ethnic Factors in MicroRNA Expression and Risk for Type 2 Diabetes*” sought to determine whether there were variations in miRNA expressions associated with the risk for type 2 diabetes in racial or ethnic groups that would imply the contribution of underlying genetic variations to miRNA-mediated gene regulation (Flowers et al.).

The miRNA regulome is a comprehensive group of regulatory components that either directly or indirectly influence miRNA expression. Its comprehension is essential for understanding miRNA functions and its role in human diseases. MiRNAs interact with various genomic elements in different omics levels making classification of miRNA-related genetic variants difficult. There are numerous kinds of genetic changes related to miRNAs, such as short and structural polymorphisms. The classification of miRNA-associated genetic variants provides a foundation for completing the nomenclature for sequence variants, organizing research, implementing methods for the integration of multi-omics data, and discovering novel functional biomarkers.
